# Prophylactic and Therapeutic Effects of MOG-Conjugated PLGA Nanoparticles in C57Bl/6 Mouse Model of Multiple Sclerosis

**DOI:** 10.34172/apb.2021.058

**Published:** 2020-07-26

**Authors:** Mehrdad Gholamzad, Hussein Baharlooi, Mehdi Shafiee Ardestani, Zeinab Seyedkhan, Maryam Azimi

**Affiliations:** ^1^Department of Microbiology and Immunology, Faculty of Medicine, Tehran Medical Sciences, Islamic Azad University, Tehran, Iran.; ^2^Department of Immunology, School of Medicine, Tehran University of Medical Sciences, Tehran, Iran.; ^3^Department of Radiopharmacy, Faculty of Pharmacy, Tehran University of Medical Sciences, Tehran, Iran.; ^4^Department of Biology, College of Basic Science, Tehran Science and Research Branch, Islamic Azad University, Tehran, Iran.; ^5^Immunology Research Center, Institute of Immunology and Infectious Diseases, Iran University of Medical Sciences, Tehran, Iran.

**Keywords:** Multiple sclerosis, Experimental autoimmune encephalomyelitis, Myelin oligodendrocyte glycoprotein, Poly (lactic-co-glycolic acid), Regulatory T cell, Immune tolerance, Biomaterials

## Abstract

***Purpose:*** Multiple sclerosis (MS) is a debilitating neuroinflammatory disorder of the central nervous system. It is believed to result from an impaired immune response against myelin components especially myelin oligodendrocyte glycoprotein (MOG). Some efforts have been made to bioconjugate the MOG peptides to tolerogenic particles like poly (lactic-co-glycolic acid) (PLGA) for treating animal models of autoimmune disorders. Accordingly, we aimed to elucidate the tolerogenic effects of MOG-PLGA particles on experimental autoimmune encephalomyelitis (EAE).

***Methods:*** PGLA nanoparticles were synthesized using water/oil/water procedure. Next, the MOG or ovalbumin (OVA) peptides covalently linked to the PLGA particles. These particles were then intravenously or subcutaneously administered to nine groups of C57BL/6 mice before and after EAE induction. The brain tissues were assessed for the infiltration of immune cells. The Tolerogenic effect of the vaccine was also assessed on the quantity of the Treg cells. Moreover, the amount of interferon-γ (IFN-γ), interleukin-10 (IL-10), and interleukin-17 levels produced by splenic lymphocytes were then quantified by ELISA.

***Results:*** Intravenous administration of PLGA_500_-MOG_35-55_ nanoparticles before EAE induction ameliorated EAE clinical scores as well as infiltration of immune cells into the brain. In the spleen, the treatment increased CD4^+^CD25^+^FoxP3^+^ Treg population and restored the homeostasis of IFN-γ, IL-10, and IL-17 (all *P* values <0.0001) among splenocytes.

***Conclusion:*** The conjugation of MOG peptides to the PLGA nanoparticles significantly recovered clinical symptoms and the autoimmune response of EAE. The MOG-PGLA particles are potentially valuable for further evaluations, hopefully progressing toward an optimal approach that can be translated to the clinic.

## Introduction

Multiple sclerosis (MS) is a prototypic neuroinflammatory disorder of the central nervous system (CNS), and is believed to result from a lack of functional harmony in immune response of autoaggressive and regulatory cells.^1,2^ Autoreactive T cells in MS have been implicated to recognize myelin proteins including myelin oligodendrocyte glycoprotein (MOG), myelin basic protein (MBP), and proteolipid protein (PLP).^3^ In contrast, CD4^+^CD25^+^Foxp3^+^ Treg cells (Tregs) specially orchestrate the quality and quantity of adaptive immune responses against self-antigens.^4-6^ However, Treg cells were found to have functional impairments in MS patients which make them unable to prevent the onset and progression of the disease.^7^ Accordingly, extended efforts have been made to reinduce the immune tolerance through administration of autoantigens; but the efficiency of this approach highly depends on the continuous and low-dose stimulation of regulatory cells that mediate immune tolerance.

A promising strategy to have a steady release of autoantigens for immune tolerance induction might be through coupling the autoantigens to solid biodegradable particles. Since poly (lactic-co-glycolic acid) (PLGA) particles have demonstrated suitable characteristics such as biocompatibility, biodegradability, and received approval from the Food and Drug Administration (FDA), they seem to be a pertinent carrier of the desirable drugs with sustained-release rate.^8^ This technology has already shown practical applicability in several settings. In particular, we have found that intravenous (i.v.) injection of MOG-conjugated PLGA before and after EAE induction, significantly inhibited proliferation of splenocytes leading to a delayed incidence of the syndrome.^9^ In other studies, PLGA-conjugated antigens depicted enhanced tolerogenic effects compared to antigens alone in nasal vaccination.^10^ More specifically, i.v. administration of PLGA-encephalitogenic peptides also indicated ameliorative outcomes in experimental autoimmune encephalomyelitis (EAE).^11^ We, therefore, set out to discover how PLGA-MOG nanoconjugates would be able to re-establish the required immune tolerance effectively in the EAE mouse model of MS.

The current study was conducted to gain more insight into the mechanism by which MOG-conjugated PLGA particles driving immune response towards the immunoregulatory response of particularly Tregs in EAE. To do so, either prophylactic or therapeutic injection methods were comparatively examined in the study.

## Material and Methods

### Mice

Inbred, 6-8 weeks old female C57BL/6 mice were purchased from Pasteur Institute of Iran (Tehran, Iran). Nine groups of mice, each containing five animals, were kept under standard housing conditions in the central animal facility at Tarbiat Modares University (TMU).

### Nanoparticle preparation

Using double emulsion solvent evaporation (W/O/W) approach at room temperature, the PLGA (Sigma-Aldrich, Gillingham, Dorset, UK) microspheres were prepared in sterile conditions as described previously.^12^ In summary, 1 mL of phosphate buffer saline (PBS, pH = 7.4) containing N-cetyl-N,N, N-trimethyl ammonium bromide (Merck, Kenilworth, NJ, USA) (0.2%, w/v) was suspended in 10 mL of 4% w/v PLGA solution in ethyl acetate, and then sonicated at 50 watt (W) for 1 min in an ice-bath. The water-in-Oil (W/O) emulsion was subsequently added into 20 mL of 2% w/v aqueous polyvinyl alcohol (88% hydrolyzed, 20.000-30.000, Achros) and mixed at high speed by mechanical stirrer at 4000 rpm. After evaporation of the organic solvent, the obtained microspheres were washed and then collected by centrifugation for 5 min at 4000 rpm. Next, the supernatant was collected and washed twice with PBS and passed through 500 to 30 kDa molecular weight cut-off membranes (Merck, Kenilworth, NJ, USA). After filtration, 3 fractions were collected and lyophilized. Molecular weight distribution of particles which passed through 500 kDa filter but precipitated on 300 kDa paper was defined as PLGA_500_. Moreover, PLGA_100_ were all particles went through 500, 300, and 100 kDa filters but could not pass from a 30 kDa filter.

### Conjugation of MOG and OVA peptides to the PLGA particles

PLGA particles were pre-activated in a mixture containing 1-Ethyl-3-(3-dimethylaminopropyl) carbodiimide (EDCI) (4 mg/mL final) and N-hydrosulfosuccinimide (Sulfo-NHS) (50 mM final) as described earlier.^13^ The activated particles were incubated on a rotator for 2 hours at room temperature. MOG_35-55_ or OVA_323-339_ (Sigma-Aldrich, Gillingham, Dorset, UK) peptides were added to the PLGA (1 mg/mL) solution and then further incubated for 72 hours in order to achieve the desired antigen coupling rates. Glycine (7 mg/mL final) was then used to saturate the unbound sites on the particles and the whole solution was incubated again for 30 min at room temperature. To remove the unbound glycine, the final solution was dialyzed in a 10-14 kDa molecular weight cut-off membrane against PBS (pH = 7.2) at 4°C overnight. Given that the final solution of antigen-conjugated PLGA could not be sterilized via filtration or UV irradiation, as it may cause peptide degradation; we, therefore, conducted the whole conjugation process under aseptic conditions and then stored the lyophilized powder at 4°C.

### Evaluation of coupling efficiency

Conjugation efficiency of the particles was indirectly calculated by measuring the amount of unconjugated proteins within the supernatant of the final solution. In summary, an aliquot of the solution was ultracentrifuged (Beckman TLA-100.3) (Beckman Coulter, Fullerton, CA, USA) at 70 000 rpm for 20-30 minutes. After collecting the supernatant, Micro BCA protein assay kit (Thermo Fisher Scientific, Waltham, MA, USA) was used to assess the free proteins according to the manufacturer’s guideline.

### Administration of PLGA-conjugated peptides to mice

Each of the investigating groups consisted of five C57BL/6 mice, and the administrations’ protocol was conducted as shown in Table 1.

**Table 1:** Administrations protocol for each individual group

**Group**	**Group name**	Day of injection*	**Dose of injection**	**Conjugate**	**Route and site of injection**
1	Test	-7	2 mg	PLGA_100_-MOG_35-55_	i.v. – Tail vein
2	Test	-7	2 mg	PLGA_500_- MOG_35-55_	i.v. – Tail vein
3	Control	-7	2 mg	PLGA_500_- OVA_323-339_	i.v. – Tail vein
4	Rechallenge	-7	2 mg	PLGA_500_-PLP_139-151_	i.v. – Tail vein
5	Test	0	2 mg	PLGA_500_-MOG_35-55_	i.v. – Tail vein
6	Test	-7	2 mg	PLGA_100_-MOG_35-55_	s.c. – Back
7	Test	-7	2 mg	PLGA_500_- MOG_35-55_	s.c. – Back
8	Control	-7	2 mg	PLGA_500_- OVA_323-339_	s.c. – Back
9	Healthy untreated group (naïve group)				

i.v.: intravenous, s.c.: subcutaneous * Reference is the day of EAE induction.

### EAE Induction

To recapitulate MS, EAE was induced in 6-8 weeks old C57/BL6 mice, using an emulsion of MOG_35-55_ peptides (1 mg/mL) and complete Freund’s adjuvant (4 mg/mL of Mycobacterium tuberculosis H37Ra (Difco, Amsterdam, Netherlands)). Moreover, mice received intraperitoneal injection of *Pertussis* toxin on the same day and 24 hours later.^14,15^ Thereafter, daily clinical symptoms of the EAE were examined using a standard scoring system ranging from 0 to 5 as follows: 0, no disease; 1, tail paralysis; 2, loss of tail tonicity and hindlimbs weakness; 3, hindlimbs paralysis; 4, hindlimb paralysis as well as forelimb paralysis; and 5, moribund.

### Histopathological analysis of brain tissue sections

The mice were deeply euthanized by ketamine-xylazine and the brain samples were carefully removed. The brains were fixed in 10 % formalin. Then, serial tissue sections from 5 to 10 μm of thicknesses were obtained from each sample, dewaxed and stained with the hematoxylin-eosin (H&E) to assess the infiltration of mononuclear cells into the brain.

### Treg cell quantification and flow-cytometry

In order to quantify Treg cells, the mice were sacrificed by cervical dislocation, and splenocytes were collected 14 days after EAE induction. Briefly, splenocytes were washed once with PBS and resuspended in 100 μL of staining buffer (PBS + 2% fetal bovine serum (FBS)). The cells were firstly stained for surface markers with anti-CD4-FITC and anti-CD25-APC (BD Biosciences, Heidelberg, Germany) according to the manufacturer’s instruction. Each sample was then fixed and permeabilized with LEUCOPERMTM (a reagent for cell permeabilizing which is supplied from Bio-Rad) (Bio-Rad, California, CA, US), based on the manufacturer’s manual. Anti-Foxp3-PE (BioLegend, San Diego, CA USA) was used for staining and the cells were evaluated by BD FACSCalibur^TM^ (BD Biosciences, Heidelberg, Germany).

### Cytokine production characterization of splenic lymphocytes

The murine spleens were removed on day 14 under sterile conditions, placed into the cell strainer separately, and homogenized. The strainer was then rinsed with 5 mL incomplete RPMI 1640 (Gibco, Gaithersburg, MD, USA) containing penicillin (100 U/mL) and streptomycin (100 μg/mL). The cell suspension was centrifuged at 500 × g for 10 min. The supernatant was discarded and the pellet was resuspended in Ammonium-Chloride-Potassium (ACK) lysing buffer. The cells were incubated at room temperature for 5 minutes, and subsequently, quenched with complete RPMI containing 10% FBS. The centrifugation was repeated at 500 × g for 10 min and the pellet was resuspended in 5 mL of complete media. A suspension of 5 × 10^5^ cells/ mL was cultivated in presence of the MOG_35-55_ (10 μg/mL) and then incubated at 37°C and 5 % CO_2_. After 72 h, the supernatant was collected and concentration of interferon-γ (IFN-γ), interleukin-10 (IL-10), and interleukin-17 (IL-17) were measured through commercial ELISA kits (Invitrogen, Waltham, MA, USA).

### Statistical analysis

All statistical analyses were conducted by SPSS software v. 24 (SPSS Inc, Chicago, IL, USA) and data were plotted using GraphPad Prism version 6.00 (GraphPad Software Inc, La Jolla, CA, USA). Each experiment was conducted in duplicate or triplicate and one-way ANOVA with Tukey’s post-hoc test as well as independent *t* test were applied to compare the differences between various treated and untreated control groups. Data are presented as means ± standard error of the mean (SEM) unless otherwise stated, and two-sided *P* values < 0.05 were considered as statistically significant.

## Results and Discussion

**Figure 1:**
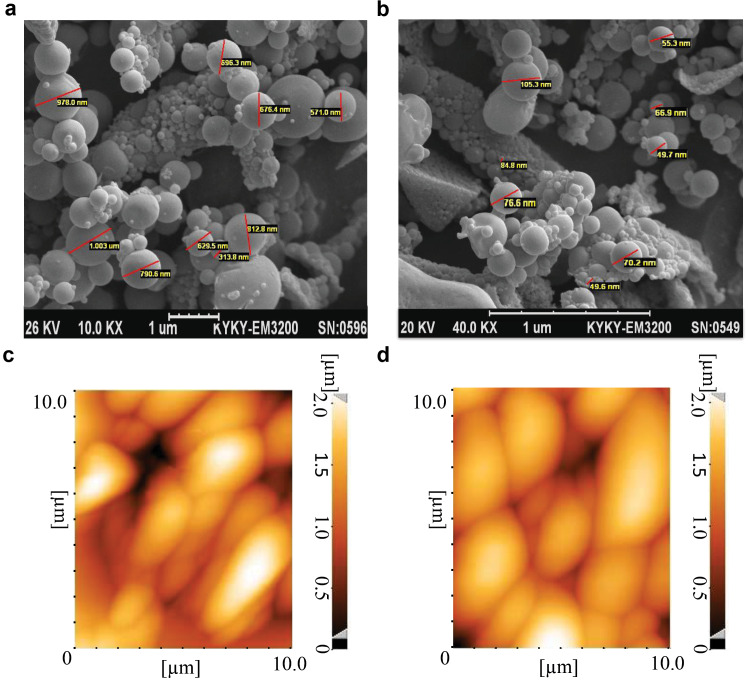
Characteristics of the PLGA particles. Scanning electron microscope micrographs of PLGA_500_ (a) and PLGA_100_ (b) particles demonstrated smooth spherical morphologies before conjugation. Additionally, *atomic-force microscopy of*PLGA_500_ particles *illustrated no morphological change as well as aggregation in*PLGA_500_ particles before (c) and after (d) conjugation. Naturally, size of the particles was increased after coupling to the MOG peptides.

In our previous study,^16^ we evaluated characteristics of the PLGA particles using scanning electron microscopy. PLGA500 and PLGA_100_ particles had spherical shape and a smooth surface (Figure 1a-b). The PLGA_500_ particles were microparticles in sizes ranging from 400 to 500 nm, while PLGA_100_ particles were nano-scale particles smaller than 100 nm. The polydisperse index (PDI) of PLGA_100_, PLGA_300_ and PLGA_500_ particles were 0.1, 0.53, and 0.85, respectively. The zeta potential of PLGA_100_, PLGA_300_, and PLGA_500_ particles was determined to be -14.1, -5.65, and -18.0, respectively. According to the dynamic light scattering (DLS) outcomes, the average size of PLGA_100_, PLGA_300_ and PLGA_500_ particles was determined as 151 nm, 389 nm and 521 nm. On the other hand, the particles integrity has not been significantly altered after conjugation of MOG_35-55_ or OVA_323-339_ with PLGA_500_ particles and were still smooth on the surface with no aggregation, as observed through *atomic force microscopy (AFM)* (Figure 1c-d). The conjugation efficiency of 9.56% and 25.85% were measured for MOG peptides conjugated to PLGA_500_ and PLGA_100_, respectively. In addition, OVA binding efficacy to the PLGA_500_ and PLGA_100_ particles was 8.59% and 24.2%, respectively.

**Figure 2:**
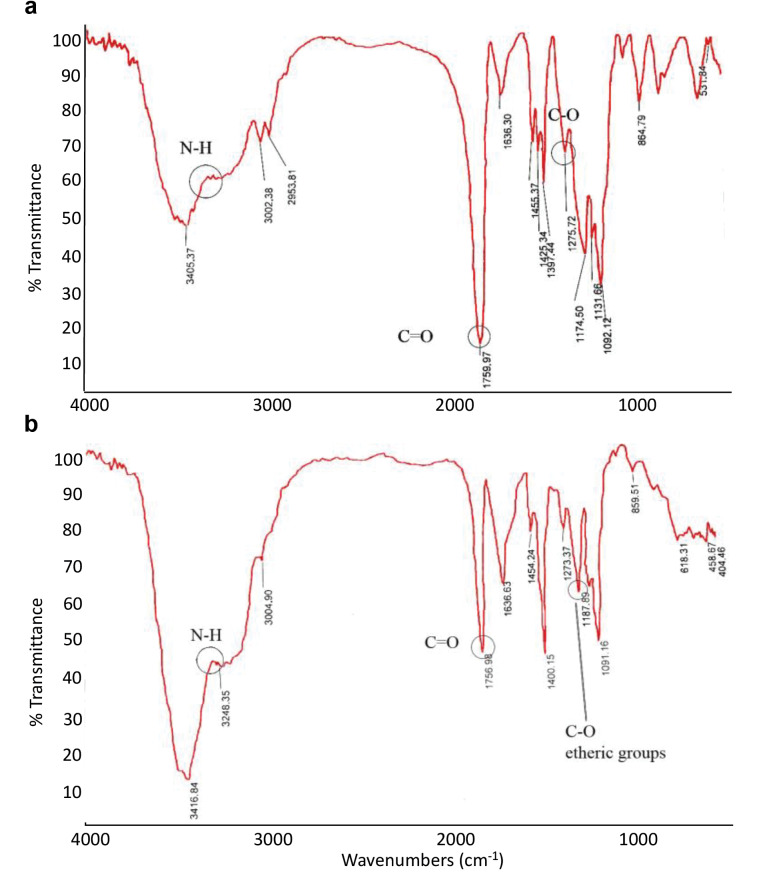
The FTIR spectrum of the PLGA_500_-MOG_35-55_ particles before (a) and after (b) conjugation to the MOG peptides. During conjugation, the decrease in wavelength peak of carboxylic group and increase of the etheric peak confirmed conjugation of PLGA to the MOG peptides. FTIR, Fourier transform infrared spectroscopy.

Fourier transform infrared spectroscopy (FTIR) technique subsequently was used to check conjugation of the peptides to the PLGA_500_. PLGA_500_ particles were placed in the device before and after coupling to the peptides, and their infrared transmittance was plotted. As shown in Figure 2, there were differences in the infrared spectrum of the nanoparticles before (Figure 2a) and after (Figure 2b) binding to the peptide, which may be due to the addition of new bands over the nanoparticles. In the plots of PLGA, the important peaks were related to those described in Table 2, before and after peptide coupling to the PLGA particles.

**Table 2:** Important peaks of conjugated and free PLGA microparticles

**Functional groups**	**Point**	**Type of vibration**	**Characteristic absorptions (cm**-^1^**)**	**Intensity**
Alkane	Before PLGA conjugation	C-H stretch	2850-3000	Strong
Carbonyl	Before PLGA conjugation	C=O stretch	1670-1820	Strong
Ether	Before PLGA conjugation	C-O stretch	1000-1300	Strong
Amine	After PLGA conjugation	N-H stretch	3300-3500	Medium

The increasing peaks for aliphatic, hydroxyl, or amide groups represented in Figure 2 of PLGA graphs compared to the PLGA_500_-MOG_35-55_ graph from 2900 cm-^1^ to 3400 cm-^1^ indicates a change in the surface of the PLGA_500_ nanoparticles following conjugation to the MOG_35-55_ peptides. Moreover, the peak reduction of carboxylic groups from the wavelength of 1759 cm-^1^ in PLGA to 1756 cm-^1^ in PLGA_500_-MOG_35-55_, as well as the peak increase of the etheric groups from 1174 cm-^1^ in PLGA to 1187 cm-^1^ in PLGA_500_-MOG_35-55_ imply the conjugation of MOG peptides to the PLGA_500_. The surface coupling can increase the peak energy of carboxylic and reduce the peak energy of the etheric groups, leading to a decrease in the peak associated with the wavelength of the carbonyl group and an increase in the peak associated with the wavelength of the etheric group.^17^

**Figure 3:**
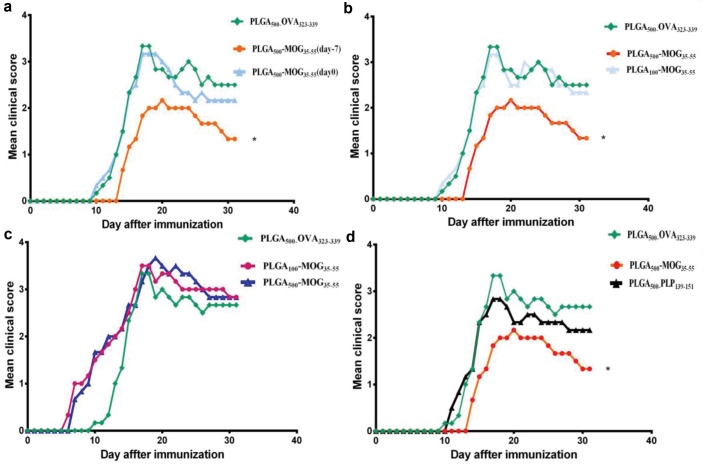
Tolerogenic effects of different PLGA particles on the clinical scores of EAE. First, we intravenously primed three groups of C57B/L6 mice with PLGA_500_-MOG_35-55_ (red line: 7 days before EAE induction, blue line: on the day of EAE induction) and PLGA_500_-OVA_323-339_ particles (control group) in order to compare prophylactic and therapeutic effects of the vaccine. Mice prophylactically treated with PLGA_500_-MOG_35-55_ (7 days before EAE induction) indicated a delayed disease onset and significant decrease of clinical scores compared to therapeutic application of the vaccine and control group (a). Next, effect of molecular size (PLGA_500_ and PLGA_100_ particles) and tolerogenic capacity of the conjugates were evaluated by prophylactic and i.v. injection of particles to three groups of mice. In this regard, we found that prophylactic PLGA_500_-MOG_35-55_ reveals significantly higher immunosuppressive effects relative to the PLGA_100_-MOG_35-55_ and control group (b). We further hypothesized if different routes of injection also affect the mean clinical scores of EAE mice. Accordingly, PLGA_100_-MOG_35-55,_ PLGA_500_-MOG_35-55_, and PLGA_500_-OVA_323-339_ were subcutaneously injected to mice, seven days before EAE induction. Our results demonstrated an early onset of EAE plus exacerbated clinical conditions for PLGA_100_-MOG_35-55_ and PLGA_500_-MOG_35-55_ treated mice compared to the control group (PLGA_500_-OVA_323-339_) (c). Finally, we aimed to confirm whether tolerance PLGA_500_-MOG_35-55_ particles was antigen-specific. Thus, PLGA_500_-MOG_35-55_, PLGA_500_-PLP_139-151_, or PLGA_500_-OVA_323-339_ particles were i.v. administered to three groups of mice seven days before the EAE induction. Our results demonstrated a specific break and reinduction of immune tolerance against MOG peptides in PLGA_500_-MOG_35-55_-treated mice compare to PLGA_500_-PLP_139-151_ and PLGA_500_-OVA_323-339_ groups (d). One-way ANOVA and Tukey’s post hoc were conducted to compare efficacy of the treatments between different groups. Data are presented as the mean ± SEM and the number of mice in each group was 4 to 6. P value less than 0.05 is summarized with *.

In the area of vaccination, non-toxic nanoparticles with low inflammatory activity and sustained release of antigens, have shown great promises to induce the proper immune responses.^18^ The current available data are controversial regarding the inflammatory activity of PLGA nanoconjugates. To investigate the tolerogenic effects of PLGA_500_-MOG_35-55_, the particles were prophylactically and therapeutically administered (i.v.) to the groups of mice on day -7 and 0 of EAE induction. Results indicated that PLGA_500_-MOG_35-55_ injection on day 0 had no significant impact on the onset and severity of the EAE. However, minor changes were observed compared to the control group received PLGA_500_-OVA_323-339_ after day 20. Interestingly, prophylactic injection of the conjugates on day -7 reduced the clinical complications (Figure 3a). It seems that the theragnostic application of these nanoparticles could partially attenuate the relapse of the disease either. The reason behind this could be possibly due to the priming condition of the mice. The prophylactic use of PLGA_500_-MOG_35-55_ provides sufficient time to strengthen the existing tolerance, while in the therapeutic manner there is less opportunity to trigger a tolerogenic response before EAE induction. Afterward, we noticed that PLGA_500_-MOG_35-55_ and not PLGA_100_-MOG_35-55_ displayed a rehabilitative impact on EAE clinical scores (Figure 3b). Indeed, the next experiments were conducted using only PLGA_500_-MOG_35-55_ particles in a prophylactic manner. Cappellano et al have also indicated that vaccination with a mixture of PLGA_500_-MOG_35-55_ and PLGA-IL-10 successfully ameliorated EAE complications in C57BL/6 mice.^19^ Compared to our analysis, they had an extra intervention of extrinsic IL-10 and consequently its tolerogenic effects. However, the two investigations were done within 40 days, and therefore, it is not possible to determine which method was more effective to induce a long-term tolerance. To predict the stability of immune tolerance, it may be advantageous to refer to the increased number of Tregs which leads to a strong tolerance in long term.^14^

As shown in Figure 3c, prophylactic and subcutaneous priming of mice with both PLGA_100_-MOG_35-55_ and PLGA_500_-MOG_35-55_ particles on day -7, exacerbated clinical conditions of the groups. This procedure also caused an early onset of the disease in the EAE mice. Thus, we found intravenous route more effective than the subcutaneous injection procedure to induce the immune tolerance. No anaphylactic reaction was observed after either s.c. or i.v. administration but the adjuvant effect of nanoparticles in subcutaneous route resulted in the exacerbation of the disease with no amelioration. On the other hand, it is worthy to note (it is worth noting) that intravenous vaccination has the potential capability to induce T cell anergy, as demonstrated by splenocyte proliferation inhibition in our previous study.^16^ Moreover, tolerance specificity of EAE induction was also evaluated when a group of mice was intravenously primed with PLGA_500_-PLP_139-151_ on day -7. Then, EAE was induced and this group demonstrated no significant improvement of clinical scores relative to the control group that received PLGA_500_-OVA_323-339_ (Figure 3d). During MS and also EAE pathogenesis, neuroinflammation permanently attracts several clones of lymphocytes that react to different epitopes of autoantigens. This phenomenon is called “epitope spreading” and points to the fact that the appropriate cure of the disease must establish the required immune tolerance against all reactive antigens.^20-22^ As a solution, exertion of homogenized CNS tissue could possibly be more clinically applicable to prevent EAE in this case. However, the present study successfully broke and then reinduced the specific immune tolerance against only MOG_35-55,_ when treatment with PLGA_500_-PLP_131-159_ nanoparticles did not affect initiation and severity of MOG-induced EAE. By contrast, Getts et al used the two epitopes PLP_139-151_ and PLP_178-191_ to demonstrate that although the induced tolerance was antigen-specific, the second epitope prevented the epitope spreading.^11^ The proximity of two epitopes in the site of lesions could possibly be the reason for their outcome. However, it needs further experimental testing in the future.

**Figure 4:**
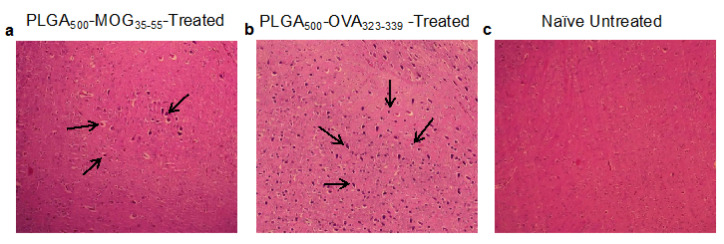
Histopathological analysis of mononuclear cells infiltrated into the brain tissues. For this purpose, brains of mice that prophylactically received PLGA_500_-MOG_35-55_ (a) or PLGA_500_-OVA_323-339_ (b) peptides, along with a healthy sample (c) were stained with hematoxylin-eosin (H&E) dyes. Arrows indicate the infiltrated cells into the different regions of the brain (hematoxylin-eosin, original magnification ×200).

In tissue level, others have previously reported infiltration of lymphocytes into the brain parenchyma during EAE pathogenesis.^23^ We, therefore, have performed H&E staining on the brain sections for each sample and found that the degree of mononuclear cell migration was significantly (markedly) reduced following the treatment with PLGA_500_-MOG_35-55_ (Figure 4a) compared to the PLGA_500_-OVA_323-339_-treated group and naïve mice (Figure 4b-c).

**Figure 5:**
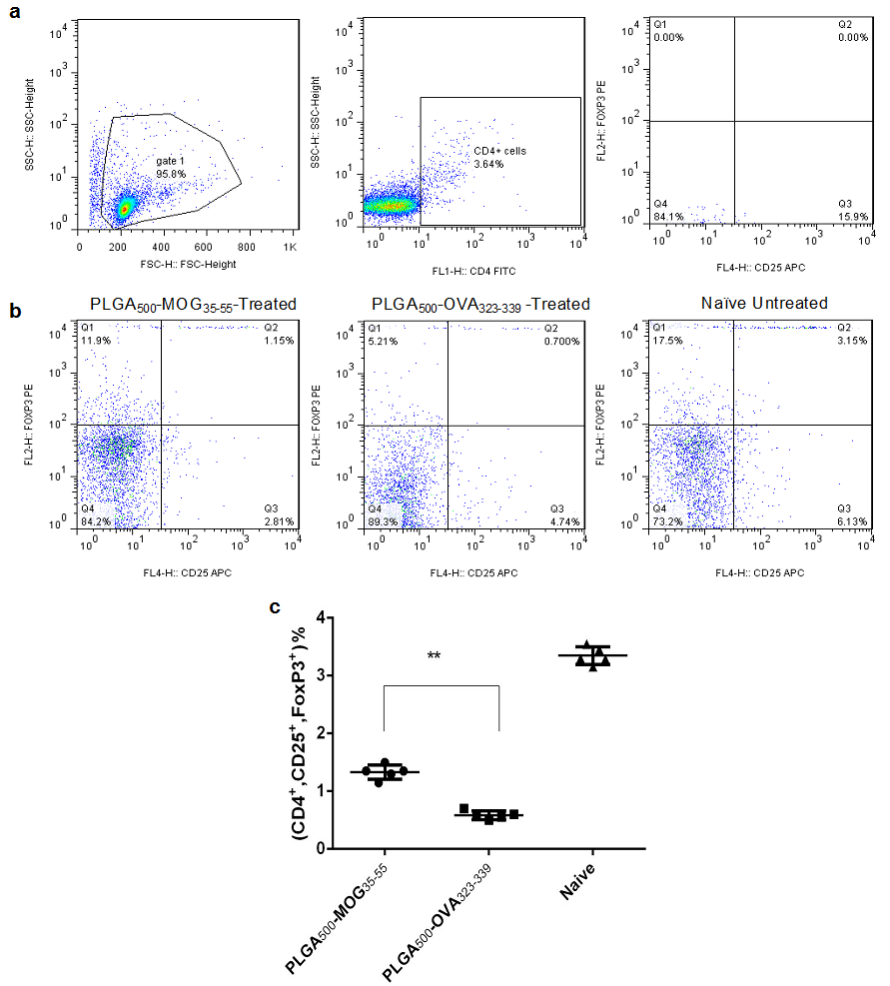
*Flow cytometric analysis of CD4*^+^*CD25*^+^*FoxP3*^+^* T cells (Tregs). Gating strategy of the splenic Tregs (a). Effect of prophylactic and i.v injection of*PLGA_500_-MOG_35-55_ and PLGA_500_-OVA_323-339_ particles on percentage of splenic Treg cells compared to a naïve mice (b). Quantification of increase in percentage of Treg cells in PLGA_500_-MOG_35-55_ compared to PLGA_500_-OVA_323-339_-treated mice (c). Independent t-test was conducted to compare PLGA_500_-MOG_35-55_, PLGA_500_-OVA_323-339_-treated. Naive group were healthy mice without any injection. Data are presented as the mean ± SEM. P value less than 0.01 is summarized with **.

To determine the potential effect of MGO-conjugated PLGA treatment on Treg frequency, three groups of mice were subcutaneously treated with PLGA_500_-MOG_35-55_ or PLGA_500_-OVA_323-339_ particles on day -7 of EAE induction. Another group was also designed as the healthy control group with no EAE induction and treatment. On day +14, the splenocytes were isolated and stimulated by MOG_35-55_ for 72 h to assess quantity of Treg cells. For analysis of CD4^+^CD25^+^Foxp3^+^ cells, lymphocytic population of isotype control was selected on forward scatter/side scatter (FSC/SSC) and then gating was applied for CD4^+^ cells. Afterward, quadrants were drawn to find percentage of CD4^+^CD25^+^FoxP3^+^ subpopulation (Figure 5a). According to the Figure 5b-c, PLGA_500_-MOG_35-55_-treated mice had significantly (*P*< 0.0001) higher count of CD4^+^CD25^+^Foxp3^+^ cells compared to those which received PLGA_500_-OVA_323-339_ particles (1.15±0.1 and 0.70±0.01, respectively).

**Figure 6:**
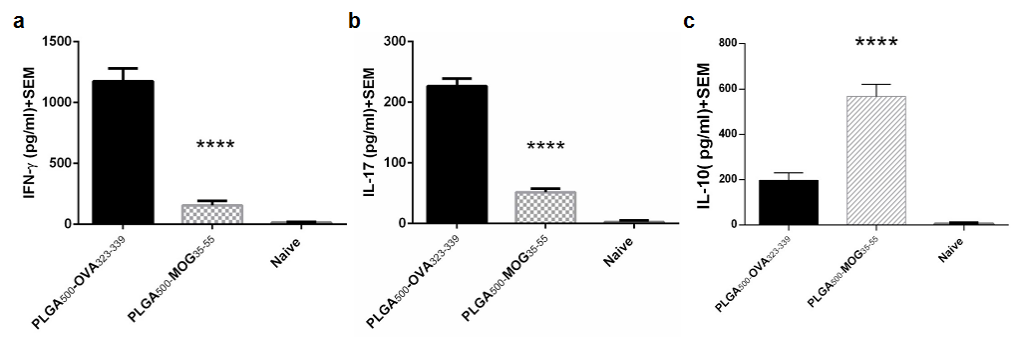
Balance of splenic cytokines in naïve mice as well as EAE-induced mice vaccinated with PLGA_500_-MOG_35-55_ or PLGA_500_-OVA_323-339_ particles. The mean concentration of IFN-γ (a), IL-17 (b), and IL-10 (c) was evaluated in the supernatant of splenic lymphocytes cultured for 72 h in presence of MOG peptides. Independent t-test were conducted to compare PLGA_500_-MOG_35-55_ -, PLGA_500_-OVA_323-339_-treated, and naïve mice together. The number of mice in each group was 5 (P≤0.00001). P value less than 0.0001 is summarized with ****.

In order to determine the immune response balance of splenocytes, the concentration of IFN-γ, IL-17, and IL-10 was measured in the supernatant of the activated cell culture. As shown in the Figure 6a-b, IFN-γ and IL-17 produced by the splenocytes of PLGA_500_-MOG_35-55_-treated mice (IFN-γ: 156±9,IL-17: 51.6±2.2 pg/mL) were significantly lower than that in PLGA_500_-OVA_323-339_-treated mice (IFN-γ: 1175±55, IL-17: 226.5±6.5 pg/mL) (both p values < 0.0001). In contrast, PLGA_500_-MOG_35-55_-treated group produced a significantly higher amount of IL-10 in culture, compared to PLGA_500_-OVA_323-339_-treated mice (567.2±22.8,196.25±12.75 pg/mL, respectively with *P* < 0.0001) (Figure 6c). Cappellano et al also reported that the PLGA_500_-MOG_35-55_ vaccine affects the balance of cytokines, by repressing either IFN-γ or IL-17 and promoting IL-10 production, which is in consensus with our results.^19^ However, uptake of PLGA particles was also shown to induce proinflammatory responses in murine macrophages and human dendritic cells.^24,25^ Moreover, we have already demonstrated that PLGA_500_-MOG_35-55_ treatment reduces proliferation capacity of activated splenocytes in EAE mouse without obvious cell toxicity,^16^ as stated by Cappellano et alin MTT assay.^19^

## Conclusion

In this study, improved clinical conditions of EAE mice were obtained in consequence of PLGA-based vaccinations, without subsequent complications reported for other treatments. Strategies exerting such particles are simple and inexpensive techniques to induce an autoantigen-specific immune tolerance. Using tolerogenic nanoparticles alongside with the immunosuppressive modules could open up new horizons towards MS treatment.

Above all, efficacy and safety of this strategy for other autoantigens of MS need to be taken for granted. Accordingly, more clinical trials for each vaccine are required to guarantee the procedure.

## Ethical Issues

The current study was conducted with approval from the Animal Ethics Committee of Tarbiat Modaress University (TMU), Tehran, Iran (thesis number: 3768).

## Conflict of Interest

The authors declare that they have no competing interests.

## Acknowledgments

We are thankful to the Department of Microbiology and Immunology facilities at Islamic Azad University-Tehran Medical Branch.

## References

[1] Hsieh CS, Zheng Y, Liang Y, Fontenot JD, Rudensky AY (2006). An intersection between the self-reactive regulatory and nonregulatory T cell receptor repertoires. Nat Immunol.

[2] Chang CC, Ciubotariu R, Manavalan JS, Yuan J, Colovai AI, Piazza F (2002). Tolerization of dendritic cells by T(S) cells: the crucial role of inhibitory receptors ILT3 and ILT4. Nat Immunol.

[3] Johnson D, Hafler DA, Fallis RJ, Lees MB, Brady RO, Quarles RH (1986). Cell-mediated immunity to myelin-associated glycoprotein, proteolipid protein, and myelin basic protein in multiple sclerosis. J Neuroimmunol.

[4] Sakaguchi S, Sakaguchi N, Asano M, Itoh M, Toda M (1995). Immunologic self-tolerance maintained by activated T cells expressing IL-2 receptor alpha-chains (CD25) Breakdown of a single mechanism of self-tolerance causes various autoimmune diseases. J Immunol.

[5] Sakaguchi S, Yamaguchi T, Nomura T, Ono M (2008). Regulatory T cells and immune tolerance. Cell.

[6] Hoffmann P, Eder R, Boeld TJ, Doser K, Piseshka B, Andreesen R (2006). Only the CD45RA+ subpopulation of CD4+CD25high T cells gives rise to homogeneous regulatory T-cell lines upon in vitro expansion. Blood.

[7] Azimi M, Ghabaee M, Naser Moghadasi A, Noorbakhsh F, Izad M (2018). Immunomodulatory function of Treg-derived exosomes is impaired in patients with relapsing-remitting multiple sclerosis. Immunol Res.

[8] Houchin ML, Topp EM (2008). Chemical degradation of peptides and proteins in PLGA: a review of reactions and mechanisms. J Pharm Sci.

[9] Gholamzad M, Ebtekar M, Shafiee Ardestani M (2017). Intravenous injection of myelin oligodendrocyte glycoprotein-coated PLGA microparticles have tolerogenic effects in experimental autoimmune encephalomyelitis. Iran J Allergy Asthma Immunol.

[10] Keijzer C, Slütter B, van der Zee R, Jiskoot W, van Eden W, Broere F (2011). PLGA, PLGA-TMC and TMC-TPP nanoparticles differentially modulate the outcome of nasal vaccination by inducing tolerance or enhancing humoral immunity. PLoS One.

[11] Getts DR, Martin AJ, McCarthy DP, Terry RL, Hunter ZN, Yap WT (2012). Microparticles bearing encephalitogenic peptides induce T-cell tolerance and ameliorate experimental autoimmune encephalomyelitis. Nat Biotechnol.

[12] Saini V, Jain V, Sudheesh MS, Jaganathan KS, Murthy PK, Kohli DV (2011). Comparison of humoral and cell-mediated immune responses to cationic PLGA microspheres containing recombinant hepatitis B antigen. Int J Pharm.

[13] Gaumet M, Vargas A, Gurny R, Delie F (2008). Nanoparticles for drug delivery: the need for precision in reporting particle size parameters. Eur J Pharm Biopharm.

[14] Getts DR, Turley DM, Smith CE, Harp CT, McCarthy D, Feeney EM (2011). Tolerance induced by apoptotic antigen-coupled leukocytes is induced by PD-L1+ and IL-10-producing splenic macrophages and maintained by T regulatory cells. J Immunol.

[15] Ghaffarinia A, Parvaneh S, Jalili C, Riazi-Rad F, Yaslianifard S, Pakravan N (2016). Immunomodulatory effect of chymotrypsin in CNS is sex-independent: evidence of anti-inflammatory role for IL-17 in EAE. Iran J Allergy Asthma Immunol.

[16] Gholamzad M, Ebtekar M, Shafiee Ardestani M (2017). Intravenous injection of myelin oligodendrocyte glycoprotein-coated PLGA microparticles have tolerogenic effects in experimental autoimmune encephalomyelitis. Iran J Allergy Asthma Immunol.

[17] Bellisola G, Sorio C (2012). Infrared spectroscopy and microscopy in cancer research and diagnosis. Am J Cancer Res.

[18] Leleux J, Roy K (2013). Micro and nanoparticle-based delivery systems for vaccine immunotherapy: an immunological and materials perspective. Adv Healthc Mater.

[19] Cappellano G, Woldetsadik AD, Orilieri E, Shivakumar Y, Rizzi M, Carniato F (2014). Subcutaneous inverse vaccination with PLGA particles loaded with a MOG peptide and IL-10 decreases the severity of experimental autoimmune encephalomyelitis. Vaccine.

[20] Waldner H, Whitters MJ, Sobel RA, Collins M, Kuchroo VK (2000). Fulminant spontaneous autoimmunity of the central nervous system in mice transgenic for the myelin proteolipid protein-specific T cell receptor. Proc Natl Acad Sci U S A.

[21] Bettelli E, Pagany M, Weiner HL, Linington C, Sobel RA, Kuchroo VK (2003). Myelin oligodendrocyte glycoprotein-specific T cell receptor transgenic mice develop spontaneous autoimmune optic neuritis. J Exp Med.

[22] Liu GY, Fairchild PJ, Smith RM, Prowle JR, Kioussis D, Wraith DC (1995). Low avidity recognition of self-antigen by T cells permits escape from central tolerance. Immunity.

[23] Stromnes IM, Cerretti LM, Liggitt D, Harris RA, Goverman JM (2008). Differential regulation of central nervous system autoimmunity by T(H)1 and T(H)17 cells. Nat Med.

[24] Nicolete R, dos Santos DF, Faccioli LH (2011). The uptake of PLGA micro or nanoparticles by macrophages provokes distinct in vitro inflammatory response. Int Immunopharmacol.

[25] Look M, Saltzman WM, Craft J, Fahmy TM (2014). The nanomaterial-dependent modulation of dendritic cells and its potential influence on therapeutic immunosuppression in lupus. Biomaterials.

